# Unveiling hidden connections: How social networks impact diversion in hospital emergency departments: An exploratory social network analysis

**DOI:** 10.1371/journal.pone.0329176

**Published:** 2025-09-02

**Authors:** Troy Francis, Maaike de Vries, Mark Fan, Reza Yousefi-Nooraie, Mathieu Ouimet, Valeria E. Rac, Patricia Trbovich

**Affiliations:** 1 HumanEra, Research and Innovation, North York General Hospital, Toronto, Ontario, Canada; 2 Institute of Health Policy, Management, and Evaluation, Dalla Lana School of Public Health, University of Toronto, Canada; 3 Program for Health System and Technology Evaluation, Ted Rogers Centre for Heart Research, Toronto General Hospital Research Institute, University Health Network, Canada; 4 Department of Public Health Sciences, University of Rochester, New York, United States of America; 5 Department of Political Science, Université Laval, Quebec, Canada; Xiamen University - Malaysia Campus: Xiamen University - Malaysia, MALAYSIA

## Abstract

Due to a lack of safeguards, controlled substances (CS) can be diverted (stolen) from healthcare facilities. While it is known that healthcare workers (HCWs) can use their social networks within the medication use process (MUP) to facilitate diversion, the specific connections between HCWs and the MUP tasks most vulnerable to diversion remain poorly understood. Social network analysis (SNA) was used to analyze social connections to identify influential relationships between HCWs and tasks susceptible to diversion.To map the social network structures of MUP tasks vulnerable to CS diversion in two Emergency Departments (EDs), identify influential tasks and HCWs, and report HCW perceptions of in-hospital diversion.This study used a mixed methods approach in the ED of two large hospitals in Toronto, Canada. Previously collected clinical observation data was used to identify tasks at risk of diversion, and cross-sectional surveys were conducted to assess HCW’s involvement in the identified vulnerable tasks. A two-mode SNA was conducted to identify connections between HCWs and tasks susceptible to drug diversion.SNA identified a circular structure across both sites, highlighting the network’s redundancy and capacity to efficiently disseminate information. Nurses were central to tasks with a higher risk of diversion. Physicians and Pharmacists had limited direct involvement in these tasks. Tasks with frequent interprofessional interactions, such as creating, following, or reviewing orders for non-admitted patients, are vulnerable to diversion due to HCWs acting on decisions made by other professionals without closely scrutinizing the details. No significant differences were observed between sites, suggesting a shared perception of diversion. The SNA results highlight the critical role of network structure in shaping vulnerability to diversion. The similarities across both sites suggest a systemic challenge across ED settings that require targeted interventions. By uncovering critical points of influence, we can better understand how diversion occurs and develop targeted interventions to prevent it.

## Introduction

Controlled substances (CSs) (e.g., opioids, methamphetamines, benzodiazepines) are essential for medical treatments, but their management poses significant challenges within healthcare facilities. Despite stringent regulations, there is compelling evidence indicating that CSs are frequently lost or stolen from hospitals [1–3]. The transfer of drugs from lawful to unlawful channels of use is referred to as ‘diversion’ and includes healthcare workers (HCWs) pilfering CS from healthcare facilities [4]. This issue underscores the necessity for enhanced checks and balances, effective triggers to signal any loss or theft promptly, and a thorough examination of the social structures within organizations that may contribute to such diversions. Although advanced monitoring systems have been developed to enhance the detection and response to irregularities, the role of social structures (patterns of social relationships among hospital staff) in facilitating the diversion of CSs has been largely overlooked [5–7]. Workplace culture, peer relationships, and hierarchical dynamics are powerful social structures that can profoundly influence behaviours [8] and create opportunities for drug diversion. For instance, the presence of collusion among staff members and gaps in oversight can undermine even the most rigorous procedural safeguards [9]. Identifying key influencers, uncovering hidden patterns of interactions, and understanding the flow of CS within hospitals is crucial for developing effective strategies to prevent diversion and ensure a safe healthcare environment [10–12].

Emergency Departments (ED) may be particularly vulnerable to diversion compared to other clinical units for several interrelated reasons and are an important clinical area to prioritize for diversion research. EDs are generally understaffed and undersourced and experience rapid patient turnover, resulting in frequent and hurried ordering and administration of CS [13–15]. This high-paced environment increases the availability of CS for diversion and reduces the time available to detect discrepancies. The ED also contains many patients not admitted to the hospital, resulting in transient documentation that is often paper-based and harder to audit. Further, multiple HCWs are involved, and there may be task sharing alongside frequent interruptions, which limits situational awareness and creates the potential for undetected diversion [4].

These characteristics of the ED also impact social relationships amongst ED staff, which can affect the opportunities for diversion throughout the medication use process (MUP; ordering, dispensing, preparing, administering, wasting, and returning) [6,7]. For example, some tasks may require a HCW to seek verification by either a peer (e.g., through witnessing or auditing) or technology (e.g., automated dispensing cabinets (ADC) using blind count backs to check inventory). If social structures compromise how thoroughly these interdependent tasks are performed (e.g., verification is skipped because staff are overly trusting of each other or are too busy to verify due to understaffing), discrepancies can arise, leading to a failure to detect various forms of diversion, including the act of stealing vials, tampering with syringes, and pilfering from waste containers. A case in point involved an ED Nurse who knowingly and intentionally entered fraudulent verbal orders for CS into the electronic medical records system on behalf of physicians who did not issue the orders. The Nurse then obtained the CS from the ADC but did not administer the drugs to the patients. Instead, the Nurse kept the substances for personal use. By building relationships with the physicians and taking advantage of the busy ED environment, the Nurse was able to gain the trust of her colleagues [16]. In this example, the Nurse was able to bypass the verification of verbal orders using her social connections and her role, which facilitated illegitimate access to CSs at multiple institutions before being caught. The social structures these tasks operate alongside have received little attention despite their important role in mediating how hospital staff interact when managing CSs. Recognizing how social structures impact the risk for diversion is important because it provides insights into the patterns and dynamics within an Emergency Department that may facilitate or hinder the misuse of CSs.

Social Network Analysis (SNA) is an approach that can help characterize how hospital staff interact when performing CS-related tasks. A social network is a social structure comprised of a set of actors (individuals, groups, or organizations) connected by 1) similarities (membership in the same professional group, demographic attributes), 2) relationships (partnerships, friendships, kinship, 3) interactions (medication-related tasks), and 4) exchanges (information) [11]. Analyzing the tasks and relationships involved in social networks within the ED can provide an understanding of where diversion risks may emerge or be reduced and how to inform interventions needed to mitigate diversion [10]. SNA characterizes social network structures by visually depicting a map (sociogram) of the network and quantifying network behaviour using metrics such as Centrality (measures of actor importance) and Cohesion (extent to which actors within a network are isolated or connected) [11,17]. In this way, SNA can identify diversion risk by describing which actors have influence (importance or affluence), social relationships, and the flows of information [18,19], which correlate with high-risk tasks. For example, SNA metrics may show close relationships between HCWs responsible for verifying medication administration; this may lead to increased trust and, therefore, reduced vigilance in adhering to verification procedures. This increases the risks that unused opioids can be simply ‘pocketed’ and documented as wasted without detection; a common method of drug diversion [2]. By mapping and contrasting multiple ED’s with unique social networks, SNA offers a way to understand how diversion risks vary and how possible mitigation strategies can be targeted.

This research applied SNA in two EDs to accomplish three objectives: (1) explore the social structure of tasks vulnerable to diversion in the MUP; (2) evaluate the influential tasks and HCWs; and (3) report HCW perceptions related to in-hospital diversion. These results can help identify social structures and tasks that promote or inhibit diversion risks within the ED.

## Methods

### Study design

We used an exploratory sequential (qual → development → QUAN) mixed methods design [20]. In phase 1, we extracted key hospital roles and CS management tasks from previously collected qualitative clinical observation data [13]. In phase 2, we developed and administered quantitative web-based cross-sectional surveys, which provided the data to generate the MUP’s social network structure and collected HCW perceptions on diversion.

### Setting

This study was conducted in the EDs of two hosptials in Toronto, Ontario, Canada: one large community academic hospital (Site 1) and one large academic hospital (Site 2). The hospitals used different automated dispensing cabinets (ADCs) and medication management systems. In both hospitals, at the time of the clinical observations, medication orders and administration were recorded using paper charts for non-admitted patients and an electronic health record (EHR) system for admitted patients.

### Data collection

This study employed a mixed methods approach to investigate the relationship between HCWs and their participation in tasks susceptible to diversion. Qualitative data was used to establish the social interactions between HCWs and tasks vulnerable to diversion in the ED. Additionally, quantitative data was collected to quantify the tasks and their frequency performed by role.

#### Phase 1: Previously collected data (clinical observations and risk assessment).

A complete description of the clinical observation methodology has been published [13,21]. The study team of health services researchers gathered clinical observation data by observing nurses, physicians, and pharmacists at both EDs. They mapped MUP activities, identified weaknesses in these processes, and described the vulnerabilities using Healthcare Failure Mode and Effect Analysis (HFMEA). HFMEA is a prospective risk analysis that involves mapping detailed process flow diagrams, systematically identifying potential failure modes, and prioritizing vulnerabilities via a structured decision-making algorithm [22]. Previously collected data (i.e., free-form notes, risk analysis scores, and identified vulnerable tasks) from sixty-three hours of clinical observations data from Site 1 (June 17 – July 14, 2018) and Site 2 (November 11–17, 2018, and January 20 – February 9, 2019) were analyzed to inform the SNA [13]. The observers took notes on how HCWs accomplished their tasks and gathered contextual information such as the unit layout, staff roles, technology usage, and locations of CS on the unit. A risk analysis was conducted by mapping each observed task and identifying potential failure modes. These failure modes were given scores based on the level of harm they could cause (negligible 1 – catastrophic 4) and the likelihood of them occurring (improbable 1 – probable 4) (15). Failure modes with sufficiently high hazard scores (calculated as harm times likelihood) were then classified as critical failure modes (CFMs) if they could either independently cause a process failure or were not easily preventable or detectable by the system, thereby increasing the risk of diversion.

#### Secondary analysis of clinical observation and risk assessment data.

To determine each unit’s social network structure, we used the results obtained from previous in-hospital diversion work [13]. These results consisted of detailed written descriptions of diversion vulnerabilities for each MUP task. We extracted the complete tasks (such as ordering medication) and sub-tasks (such as initiating or creating the order) of each stage of the MUP to identify the social interactions (HCW connections) involved in each unit.

A modified framework analysis was used to analyze the extracted tasks, sub-tasks, and descriptions of the social interactions involved. One reviewer (TF) systematically analyzed free-form notes in a deductive manner to discern the roles and interactions associated with each task. An example of an interaction is as follows: in the ordering of CS, the physician, in the prescriber role, communicates verbally with the nurse that an order has been written for the patient [13]. This task represents a social interaction between a physician in the role of a medication prescriber and a nurse in the role of a medication administrator [23]. A clinical observer (MDV) reviewed the codes and identified any discrepancies in the coding of social interactions. The research team (TF, MDV, MF) resolved these discrepancies through discussion. These codes were then used to generate a preliminary understanding of the social networks that are vulnerable to diversion in each task. As the clinical observations were task-focused, not all social interactions among roles were observed. This highlighted the need to gather information directly from HCWs.

#### Phase 2: Social network survey.

We used the qualitative data to create the survey questions for phase 2, specifically targeting HCWs involved in tasks that are prone to diversion. We conducted a cross-sectional survey of ED staff to understand the relationship between HCWs and their involvement in tasks vulnerable to diversion. The survey provided information about the number of HCWs performing these tasks, their frequency, and the combination of tasks being performed.

**Participants:** Staff Physicians, Registered Nurses, Registered Practical Nurses, Nurse Practitioners, Physician Assistants, and Clinical Pharmacists who interacted with CSs (within the ED MUP) and provided consent were purposively recruited to complete web-based cross-sectional surveys. Staff provided informed consent through an online framework that included a detailed study information sheet and an electronic consent form. Participants reviewed the information sheet and indicated their consent by electronically signing the form prior to beginning the survey. This process ensures informed, written (electronic) consent was obtained from all participants.

**Data Collection and Materials:** A matrix table outlining person-by-task data was collected to capture data on as much of the target population as possible (approximately 300 HCWs within ED). We aimed to enroll 120 participants, 60 at each institution. A response rate of 75% was considered adequate to calculate social network properties [24]. A voluntary link to participate in the web-based survey was sent by the unit/department managers to eligible staff, with up to two email reminders. Survey responses were collected between December 2022 – July 2023 and managed using REDCap (Research Electronic Data Capture) electronic data capture tools [25]. REDCap is a secure, web-based software platform that supports data capture for research studies [26]. The survey had three main sections (S1 Appendix). The first section collected data on HCW attributional characteristics (age, sex, clinical unit, role, years of experience, and workload). The second section collected information reflecting vulnerable tasks to diversion. Each HCW was asked to report whether they participated in tasks vulnerable to diversion and how often (daily, weekly, monthly, and every 2–3 months). The final section collected data on HCW perceptions regarding diversion and possible safeguards. The boundaries of each social network studied were confined to the ED and the HCWs within them [17].

### Ethics

Ethics approval was obtained from participating hospitals (2023-0221-409 and 22−5275). All data were anonymized. Survey responses were replaced with unique study identifiers (P-01, N-01, Ph-01, etc.) and stored according to the Research Ethics Board’s requirements.

### Data Analysis–Social network analysis

Using the responses collected in the survey, a two-mode SNA was performed for each site’s ED. Two-mode networks, also called affiliation networks, map the implicit relationship between two sets of nodes, and connections only exist between nodes of the different sets, not within the same set. In this study, the sets of nodes reflect HCWs and tasks. The connections indicated which HCWs (such as Physicians, Nurses, or Pharmacists) participate in specific tasks vulnerable to diversion. It’s important to note that in a two-mode network, no direct connections exist between nodes of the same type (i.e., HCW to HCW or task-to-task ties). The risk scores from the phase 1 data (only apply to MUP tasks) represented a task’s level of harm and likelihood of occurring and were categorized into low (1–6), medium (7–11), or high (12–16) vulnerability ratings.

The two ED networks were then compared by a) qualitatively assessing network structure and b) quantifying key SNA metrics. The structure of the network diagrams is influenced by the position of HCWs and the tasks they participate in. The connections between HCWs and tasks were also weighted based on the frequency in which these tasks were performed by each HCW, e.g., daily (4), weekly (3), monthly (2), and every 2–3 months (1). Key quantitative SNA metrics were calculated to measure node-level (centrality) and network-level (cohesion) indices. SNA metrics were also used to identify influential nodes: HCW or task nodes that significantly impact the overall structure, information flow, or network behaviour. Weighted degree and eigenvector centrality were calculated for each node (HCW and task) to determine centrality. Normalization was used to account for network size and connection differences to compare the two sites. Normalized network metrics were bounded between 0 (no contact) and 1 (maximal contact). Cohesion was assessed to look at the total level of connectivity within the whole network (global clustering coefficient) and node redundancy to understand the importance of a HCW in maintaining the network’s connectivity. To summarize, Table 1 outlines important network terms and measures for studying HCW and task networks in more detail.

**Table 1:** SNA metrics.

SNA Metric		Measure	Interpretation	Diversion Example
Centrality (Quantifies connectedness and ‘influence’ of individual nodes)	Weighted Degree Centrality (normalized)*	Identifies the number of connections a node has while accounting for the strength of the tie [31].	**HCW:** high degree reflects involvement in *many tasks* vulnerable to diversion **Task:** A high degree reflects the involvement of *many HCWs*	A HCW involved in many tasks may be able to manipulate several steps in the MUP to create the appearance of a legitimate transaction to obscure diversion. A task performed by many Nurses, Physicians, Pharmacists, or a combination may signify that multiple HCWs oversee it.
	Weighted Eigenvector Centrality (normalized)*	Identifies how connected a node is to other highly connected nodes [32].	**HCW:** high eigenvector reflects involvement in *many tasks* vulnerable to diversion, which are influential. **Task:** high eigenvector reflects that a HCW is connected to tasks that are, in turn, assigned to many other HCWs.	A Nurse involved in many tasks can abuse their connection to other influential tasks in the network to obscure diversion (e.g., due to the constant turnover of HCWs, it is laborious to investigate discrepancies) Adding oversight from HCWs not involved in other vulnerable influential tasks can act as a stop-gap measure to mitigate the risk of diversion. E.g., A Pharmacist who reviews CS order logs and does not have other tasks in the MUP is more accountable for detecting a discrepancy.
Cohesion (Quantifies connectivity within the network)	Global Clustering Coefficient (GCC)	Quantifies the overall level of connectivity in a network (across both HCW and Task nodes) [33].	*High* GCC reflects many connections between HCWs and tasks leading to a more secure network. *Low* GCC implies HCWs are more likely to work alone or have few peers monitoring their work on any given task.	A low global clustering coefficient can reflect a lack of connections between professional groups, leading to social boundaries that reflect subgroups that limit information sharing and increase the risk of diversion due to less oversight across professional groups.
	Node Redundancy	Measures a node’s importance in maintaining the network’s connectivity [30].	Identifies HCWs that are critical or redundant in the units MUP.	Highly redundant networks provide resilience and increased security by maintaining multiple connections among HCWs, ensuring continuity even if some nodes are removed.

* Data is normalized to allow for network comparisons. Degree centrality: Dn = D/ (n − 1)), where Dn is the normalized value, D is the raw degree value, and *n* is the number of nodes in the network. Eigenvector centrality is normalized by scaling by the maximum value of the eigenvector.

Survey demographic and HCW perception data were analyzed descriptively using counts and percentages, and differences between sites were assessed using Pearson’s Chi-squared test. All statistical analyses were performed using R Software [27] and SNA metrics were calculated using packages “igraph”, “migraph”, and “tnet” [28–30].

## Results

Participant characteristics are displayed in Table 2. There were differences in participant characteristics between the sites regarding ‘Sex’ (p = 0.047), ‘Age’ (p = 0.027), ‘Primary Role’ (p = 0.011), and ‘Workload’ (p = 0.024). Site 1 had more female healthcare workers, younger staff, more nurse involvement, and a larger part-time workforce. Site 1 had limited Physician involvement compared to Site 2 primarily because their ED was significantly burdened with COVID-19 during recruitment, resulting in limited physician respondents. Only HCWs who reported involvement in tasks vulnerable to diversion within the network survey were included in the SNA. In Site 1, 59 HCWs were included, while in Site 2, 57 HCWs were included in the SNA.

**Table 2:** Participant characteristics by site.

	Site 1 (N = 62)	Site 2 (N = 59)	p-value
**Sex**			**0.05** ^1^
- Male	10 (16.1%)	18 (31.6%)	
- Female	52 (83.9%)	39 (68.4%)	
**Age**			**0.03** ^1^
− 18 - 25	15 (24.2%)	4 (7.0%)	
− 26 - 35	34 (54.8%)	28 (49.1%)	
− 36 - 45	8 (12.9%)	14 (24.6%)	
− 46 - 59	4 (6.5%)	10 (17.5%)	
− 60+	1 (1.6%)	1 (1.8%)	
**Years in Clinical Area**			0.64^1^
- < 1 year	11 (17.7%)	9 (15.8%)	
− 1–5 years	28 (45.2%)	21 (36.8%)	
− 6–10 years	10 (16.1%)	10 (17.5%)	
− 11–15 years	7 (11.3%)	6 (10.5%)	
- > 15 years	6 (9.7%)	11 (19.3%)	
**Primary Role**			**0.01** ^1^
- Registered Nurse	50 (80.6%)	32 (56.1%)	
- Physician – Staff	3 (4.8%)	17 (29.8%)	
- Registered Practical Nurse	1 (1.6%)	4 (7.0%)	
- Registered Nurse – Charge Nurse	2 (3.2%)	1 (1.8%)	
- Registered Nurse – Clinical Coordinator	1 (1.6%)	0 (0.0%)	
- Registered Nurse – Nurse Educator	1 (1.6%)	0 (0.0%)	
- Pharmacist – Clinical	1 (1.6%)	1 (1.8%)	
- Physician Assistant	0 (0.0%)	1 (1.8%)	
- Nurse Practitioner	0 (0.0%)	1 (1.8%)	
- Other	3 (4.8%)	5 (8.8%)	
**Years of Experience in Role**			0.33^1^
- < 1	8 (12.9%)	6 (10.5%)	
− 1 - 5	28 (45.2%)	21 (36.8%)	
− 6 - 10	16 (25.8%)	12 (21.1%)	
− 11 - 15	5 (8.1%)	5 (8.8%)	
− 16 - 20	3 (4.8%)	5 (8.8%)	
- > 20	2 (3.2%)	8 (14.0%)	
**Workload**			**0.02** ^1^
- Full-time	47 (75.8%)	46 (80.7%)	
- Part-time	12 (19.4%)	3 (5.3%)	
- Casual	3 (4.8%)	8 (14.0%)	
**Shift**			0.76^1^
- Rotating days and nights	52 (83.9%)	45 (78.9%)	
- Days only	8 (12.9%)	9 (15.8%)	
- Nights only	2 (3.2%)	3 (5.3%)	
**SNA Metrics**			N/A
HCW Nodes	59	57	
Task Nodes	15	15	
Edges	805	552	
Global clustering Coefficient	0.99	0.95	

^1^Pearson’s Chi-squared test.

### Social network analysis

**Fig 1:**
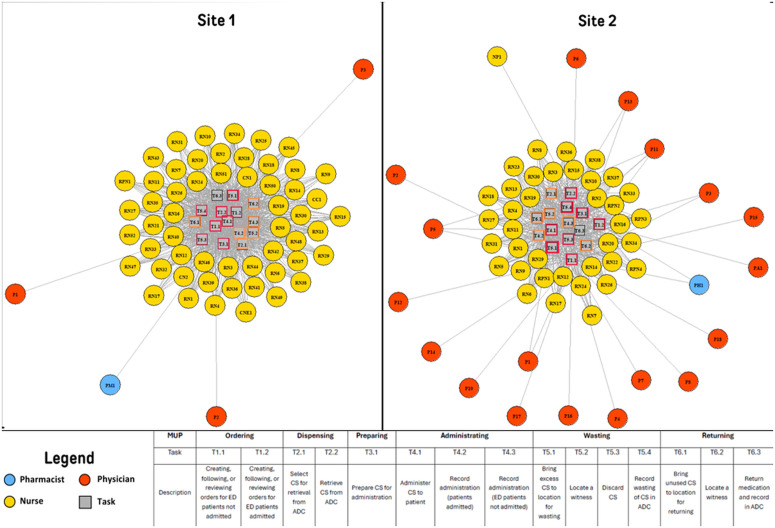
Two-Mode Network Diagram of Site 1 & 2 ED. *Red denotes tasks with a high vulnerability rating; Orange denotes tasks with a medium vulnerability rating; Black denotes tasks with a low vulnerability rating.

The SNA results are organized into three categories: the influence of HCWs within the network, influential tasks, and the network structure, which includes the arrangement of nodes and connections. Fig 1 depicts the two-mode network diagram of Sites 1 and 2 (weighted edges are not depicted), with circular nodes representing Physicians (red), Nurses (yellow), and Pharmacists (blue) and square nodes (grey) depicting the tasks vulnerable to diversion they participate in.

### Influential healthcare workers

Across both sites, Nurses (Registered Nurses and Registered Practical Nurses) play a pivotal role in the network. They are the most central actors with a high average degree centrality (Table 3), indicating their access and reach in many tasks vulnerable to diversion. Nurses are also highly involved in influential tasks (tasks that significantly impact the structure, dynamics, and flow of information in the network, Table 3). This can be beneficial, as it adds redundancy and strengthens protection within the network. However, it also poses a risk, as nurses may be able to leverage their involvement in these key tasks to facilitate diversion. Compared to Nurses, Physicians and Pharmacists were situated on the network’s periphery at both sites (Fig 1), as their involvement in vulnerable tasks was primarily limited to creating and reviewing orders for CS (T1.1, T1.2). Despite their peripheral network position, Physicians and Pharmacists are integral to ensuring that CS are used for legitimate purposes and not diverted. In particular, Physicians were the primary HCWs in the network responsible for creating orders and were also involved in selecting CS for retrieval (T2.1) and administering CS to patients (T4.1). Both sites had dedicated ED Pharmacists who played key roles in reviewing orders (T1.1 and T1.2) to prevent misuse by identifying discrepancies in ordering and dispensing. This oversight by Physicians and Pharmacists is crucial for maintaining the integrity of CS management and preventing diversion. S1 and S2 Tables provide the individual centrality statistics for HCWs from Sites 1 and 2, respectively. Many Nurses at both Site 1 (73%, 43/59) and Site 2 (42%, 24/57) reported involvement in every task, resulting in the highest Degree (1.00) and eigenvector (0.20) centrality. This indicates that a large proportion of influential Nurses are well-connected and knowledgeable about all the processes in the system, adding redundancy to the network. However, this high centrality also poses a risk: it may enable nurses to divert CS themselves, or make them targets for others who recognize that their extensive involvement in key tasks could be exploited for diversion.

**Table 3:** Mean HCW centrality statistics.

SNA Metric	Site 1 (N = 62)	Site 2 (N = 59)	p-value
Mean Nurse Degree Centrality (SD)	0.97 (0.07)	0.92 (0.17)	**0.05** ^2^
Mean Physician Degree Centrality (SD)	0.07 (0.00)	0.10 (0.06)	0.40^2^
Mean Nurse Eigenvector Centrality (SD)	0.19 (0.01)	0.22 (0.04)	**< 0.001** ^2^
Mean Physician Eigenvector Centrality (SD)	0.01 (0.00)	0.03 (0.01)	**0.05** ^2^

^2^Linear model ANOVA. Mean Pharmacists’ statistics were not calculated as there was only 1 per site.

### Influential MUP tasks

Various factors influence how likely a task is to contribute to diversion. First, the task’s diversion vulnerability rating (low, med, high) reflects its inherent risk. Second, the degree centrality of a task can signal its security against diversion – a high degree may offer better oversight through increased involvement from multiple HCWs but could also increase exposure to manipulation (e.g., become difficult to attribute responsibility for any discrepancies or issues that arise) if appropriate measures are not in place. Understanding degree centrality allows organizations to identify critical tasks that require additional safeguards and monitoring, ensuring that the benefits of oversight are maximized while mitigating the risks associated with increased exposure. Third, the task‘s eigenvector centrality indicates its protection against diversion through redundancy (e.g., HCW is involved in a task assigned to many other HCWs, providing oversight to mitigate the risk of diversion). Table 4 shows the task’s vulnerability rating and network descriptive statistics. Each site’s network was primarily composed of tasks with a high vulnerability rating for diversion (8/15 tasks). Node T1.1 has a high vulnerability rating and degree centrality, making it a highly influential task within the network, involving Physicians ‘creating’ orders, Nurses ‘following’ orders, and Pharmacists ‘reviewing’ orders. This task benefits from its multiple interprofessional connections, enhancing the detection of diversion through the involvement of various disciplines. However, these interprofessional connections can also complicate accountability, making it challenging to identify the source of the problem if proper tracking and reconciliation processes are lacking.

**Table 4:** Summary of network centrality statistics related to tasks.

		Degree Centrality		Eigenvector Centrality	
Node	Vulnerability Rating	Site 1	Site 2	Site 1	Site 2
**T1.1** (Creating, following, or reviewing orders – not admitted)	**High**	**0.97**	**0.95**	**0.37**	**0.38**
T1.2 (Creating, following, or reviewing orders -admitted)	High	0.88	0.61	0.35	0.34
T2.1 (Select CS for retrieval from ADC)	Medium	0.83	0.53	0.34	0.31
T2.2 (Retrieve CS from ADC)	High	0.86	0.60	0.35	0.35
T3.1 (Prepare CS for administration)	High	0.93	0.67	0.37	0.38
T4.1 (Administer CS to patient)	High	0.93	0.68	0.37	0.38
T4.2 (Record administration – admitted)	Medium	0.86	0.63	0.35	0.37
T4.3 (Record administration – not admitted)	Medium	0.93	0.65	0.37	0.38
T5.1 (Bring excess CS to location for wasting)	High	0.92	0.65	0.37	0.38
T5.2 (Locate a witness)	Medium	0.93	0.65	0.37	0.38
T5.3 (Discard CS)	High	0.92	0.65	0.37	0.38
T5.4 (Record wasting of CS in ADC)	High	0.93	0.63	0.37	0.37
T6.1 (Bring unused CS to location for returning)	Medium	0.90	0.61	0.36	0.36
T6.2 (Locate a witness)	Medium	0.93	0.56	0.37	0.34
T6.3 (Return medication and record in ADC)	Low	0.92	0.61	0.37	0.36

### Network topology

Both networks are arranged in a circular ring topology (Fig 1). This structure allowed information to quickly spread throughout the network due to the many connections across HCWs and tasks reflecting the high global clustering coefficient (Table 2). This clustering reflects a cohesive network and a protective effect that can quickly identify and communicate discrepancies in the accounting and management of CS across the MUP. Additionally, this structure reflects redundancy, which is able to keep the network connected and ensures continuity. S3 and S4 Tables provide the individual redundancy scores for HCWs from Sites 1 and 2. Within both sites, numerous Nurses had redundant ties to tasks within the MUP, ensuring that workflow processes are maintained even if a HCW is unavailable (e.g., doesn’t show up for a shift, forgets to perform a task, or performs the task incorrectly). This redundancy is essential for influential tasks such as T1.1, which involves a collaborative effort among multiple HCWs (Physicians, Nurses, and Pharmacists), thereby enhancing overall resilience in the workflow. That is, these redundant ties serve as an embedded diversion mitigation strategy, ensuring that multiple HCWs are involved in ordering, filling, and administering the correct doses, thereby helping to identify discrepancies.

### Healthcare worker perceptions of diversion

Table 5 displays the perceptions of HCWs of in-hospital diversion. No significant differences were observed between the different sites, reflecting a general consensus on perceptions regarding diversion. We received a total of 119 responses from our 120 enrolled participants (99% response rate). Although the response rate was high, we acknowledge the potential for non-response bias. It is possible that those who chose not to participate may hold systematically different views or experiences related to diversion. On both units, most staff were uncertain whether diversion was an issue in Canadian hospitals (Q1) and in their own unit (Q2). The majority of HCWs were unsure whether most drug diversion goes undetected (Q3) but were confident (69.7%) in the safeguards in place (Q4) on their unit to prevent diversion. Staff reported that they would talk to each other about suspicious activity (Q5) and report any incidents (Q6) (68.1%). Most staff believed every clinical area had an equal risk of diversion (58%), with 28.6% of HCWs thinking that the emergency department had the highest risk of CS loss (Q7).

**Table 5:** Perception of HCWs regarding in-hospital diversion.

	Site 1 (N = 62)	Site 2 (N = 57)	Total (N = 119)	p-value
**Q1. Diversion is a problem within Canadian hospitals.**				0.85
- Agree	17 (27.4%)	18 (31.6%)	35 (29.4%)	
- Uncertain	37 (59.7%)	33 (57.9%)	70 (58.8%)	
- Disagree	8 (12.9%)	6 (10.5%)	14 (11.8%)	
**Q2. Diversion is a problem within your unit.**				0.68
- Agree	1 (1.6%)	2 (3.5%)	3 (2.5%)	
- Uncertain	37 (59.7%)	33 (57.9%)	70 (58.8%)	
- Disagree	30 (48.4%)	30 (52.6%)	60 (50.4%)	
**Q3. “Most” drug diversion within hospitals goes undetected.**				0.89
- Agree	13 (21.0%)	14 (24.6%)	27 (22.7%)	
- Uncertain	37 (59.7%)	33 (57.9%)	70 (58.8%)	
- Disagree	12 (19.4%)	10 (17.5%)	22 (18.5%)	
**Q4. How confident are you in the effectiveness of the safeguards used on your unit in mitigating diversion?**				0.49
- Confident	45 (72.6%)	38 (66.7%)	83 (69.7%)	
- Uncertain	17 (27.4%)	18 (31.6%)	35 (29.4%)	
- Not at all confident	0 (0.0%)	1 (1.8%)	1 (0.8%)	
**Q5. Would you tell a co-worker if you noticed suspicious activity regarding possible diversion?**				0.08
- Yes	36 (58.1%)	45 (78.9%)	− 81 (68.1%)	
- Maybe	16 (25.8%)	9 (15.8%)	− 25 (21.0%)	
- Uncertain	7 (11.3%)	1 (1.8%)	− 8 (6.7%)	
- Unlikely	2 (3.2%)	2 (3.5%)	− 4 (3.4%)	
- No	1 (1.6%)	0 (0.0%)	− 1 (0.8%)	
**Q6. Would you report the individual?**				0.15
- Yes	41 (66.1%)	40 (70.2%)	− 81 (68.1%)	
- Maybe	14 (22.6%)	15 (26.3%)	− 29 (24.4%)	
- Uncertain	7 (11.3%)	1 (1.8%)	− 8 (6.7%)	
- Unlikely	0 (0.0%)	1 (1.8%)	− 1 (0.8%)	
**Q7. What clinical area do you think has the highest risk of diversion?**				0.38
- Equal risk across clinical areas	36 (58.1%)	33 (57.9%)	− 69 (58.0%)	
- Emergency Department	18 (29.0%)	16 (28.1%)	− 34 (28.6%)	
- Inpatient Pharmacy	5 (8.1%)	3 (5.3%)	− 8 (6.7%)	
- Intensive care unit	1 (1.6%)	4 (7.0%)	− 5 (4.2%)	
- Post-anesthesia care unit	0 (0.0%)	1 (1.8%)	− 1 (0.8%)	
- No response	2 (3.2%)	0 (0.0%)	− 2 (1.7%)	

## Discussion

We described the network structure of two EDs and identified influential HCWs, tasks, and their perceptions on diversion across both sites.

### Influential healthcare workers

Nurses were shown to be the most central actors within the network, demonstrating a high level of involvement in influential tasks that are particularly vulnerable to diversion. Their extensive connectivity equips them with knowledge about all processes in the system. However, this centrality also presents a potential risk; the more connected they are, the more susceptible they may be to targeted diversion attempts, mainly if adequate oversight mechanisms are not in place. In contrast, Physicians and Pharmacists occupy a more peripheral position within the network due to their relatively limited involvement in these tasks. Physicians primarily fulfill the essential functions of creating orders and administering CS to patients. Dedicated ED Pharmacists play a critical role in safeguards against diversion by identifying discrepancies in both ordering and dispensing of CS, serving as an oversight against diversion. The contrasting positions of Nurses, Physicians, and Pharmacists within the network underscores the complexity of drug diversion prevention within the ED setting. Given the interconnected nature of their roles, there is a pressing need for enhanced communication and collaborative strategies among these professionals. A lack of communication and collaboration among healthcare workers was evident in the case of the ED nurse who diverted controlled substances through fraudulent verbal orders [16]. The use of SNA would have highlighted the lack of connections in the network, which represented redundancy and oversight. The absence of these connections allowed the nurse to exploit her position and enter fraudulent orders into the electronic medical record system without being noticed by her peers.

### Influential MUP tasks

Node T1.1 was identified as an Influential task due to its high vulnerability rating and the diverse HCW roles involved. The involvement of multiple HCWs in T1.1 distributes responsibilities for monitoring and oversight among several individuals, which can be advantageous for maintaining workflows. However, this distribution can also create ambiguity in accountability. The challenge is further exacerbated when HCWs, such as nurses, routinely act on decisions made by others (e.g., a nurse routinely filling an order from an ED Physician), without thoroughly verifying the appropriateness of the order. This lack of scrutiny can create an opportunity for diversion. For instance, if a nurse receives an order from a physician and simply fills it without questioning or verifying the rationale, this could lead to inappropriate dispensing of CSs. If there’s a diversion attempt (e.g., a physician ordering to obtain CS for non-medical use), the nurse may not realize they are participating in a problematic action due to the trust placed in the physician’s decision. The initial ordering process shapes the subsequent work of the other roles, underscoring its importance within the MUP and the need for both technological and social mitigation efforts. Additionally, when discrepancies do arise without formal interprofessional connections to help investigate, these incidents can be chalked off as unexplained losses [1]. For instance, a nurse was able to pilfer CSs from her hospital’s medical supply cabinet. Despite discrepancies in her drug inventory records, her nursing colleagues covered for her, inadvertently allowing her to continue diverting [34]. By applying SNA and establishing interprofessional relationships within the network, oversight of verification and monitoring tasks can be improved to reduce diversion.

### Network topology and vulnerable workflow processes

Both sites used a circular ring network topology, showcasing the network’s capacity to efficiently disseminate information and maintain connectivity. This topology emphasizes the protective impact and resilience of redundant structures [35], ensuring workflow continuity even if a HCW fails to perform a task accurately or at all. In cases where a key process has been missed or performed incorrectly, discrepancies can be promptly detected and communicated throughout the network. However, these structures also have some attributes, such as being decentralized (no single central node; all nodes are equally important in maintaining structure) and insular (resistance to external change), which may make them vulnerable to diversion and difficult to restructure [36]. SNA can be used to restructure the ED network to limit the potential for undetected diversion. Through the inclusion of a centralized Information Technology (IT) system, technology could be used to support the redesign of network patterns. By changing from a decentralized system and removing select redundant ties and replacing them with a centralized technology, the additional HCWs could be relocated to other high-vulnerability areas of the network which require oversight [37]. For example, Samarth *et al* [38] used SNA to improve the throughput of their surgical patients. They discovered a hierarchical network structure within their post-anesthesia care unit, where the Charge Nurse served as a bottleneck by controlling all communication downstream, causing delays for patients. As a result, they restructured their organizational network to a more democratic system where an IT system coordinated the team, reducing reliance on the Charge Nurse and improving patient throughput [38].

### Perceptions on diversion

Limited information exists in the literature regarding HCWs’ perceptions or attitudes towards in-hospital diversion. This study assessed HCWs’ perceptions of diversion and found no significant differences in responses between different sites, indicating a consensus. The data rely on self-reported perceptions, which may be influenced by social desirability, fear of judgement, or lack of awareness. These biases could lead to an underestimation of the frequency of diversion or an overestimation of the effectiveness of safeguards. or apathy towards drug diversion. Hospital policies are instrumental in shaping diversion risks by influencing how controlled substances are stored, accessed, tracked, and wasted. Policies that lack clear accountability structures or rely heavily on individual vigilance can increase vulnerabilities, whereas standardized procedures, independent double-checks, and routine audits can mitigate risks. While diversion prevention policies likely differ across sites, our findings did not show clear evidence that these differences impacted results. Most respondents across all sites reported that safeguards on their units were adequate. However, perceived adequacy does not necessarily reflect objective effectiveness, and future work should explore how variation in specific policy interventions influences actual diversion outcomes and network vulnerabilities. While both the United States and Canada are among the largest consumers of opioids globally, Canadians appear to be less certain about the prevalence of diversion when compared to their American counterparts. According to a bi-annual report on diversion perceptions among American hospital executives and HCWs, 95% agree that drug diversion incidents occur in hospitals, 79% believe that “most” drug diversions go undetected, and 66% are somewhat/not confident in the effectiveness of their Drug Diversion Program [39]. In contrast, 59% of the HCWs in our study expressed uncertainty about the extent of diversion as a problem in Canadian hospitals, indicating a need for increased education on diversion within these institutions. Additionally, only 23% of HCWs in our study agreed that ‘most’ diversion goes undetected, with a significant portion (59%) being uncertain, further highlighting the necessity for enhanced diversion education for staff. Finally, we reported that 70% of respondents were confident in the effectiveness of the safeguards in their unit, revealing a notable disparity from the 66% of executives/HCWs who lack confidence in their unit’s safeguards. This emphasizes a clear difference in the safeguards used, the understanding of these safeguards, and the knowledge or accountability regarding CS loss within each unit. Furthermore, only 68% of respondents indicated that they would report an individual if they observed suspicious activity. To strengthen network security from diversion, it is critical to provide more education and services to raise awareness among HCWs about the severity of diversion and to offer avenues to report incidents without the fear of prosecution.

## Limitations and future research

There are limitations to consider when interpreting our study, as some interpretations are propositional. First, our study used data previously collected in 2018 from direct observations in the ED before the COVID-19 pandemic, which may have impacted workflow processes. Second, only tasks rated via the HFMEA as vulnerable to diversion (critical failure modes) were modelled, not every MUP task in the unit. Thirdly, SNA cannot account for the totality of an ED network due to HCWs and processes continually being altered over time due to institutional turnover [40]. Fourthly, our application of SNA metrics to diversion (Table 1) is an innovative proposition that should be tested and validated in various EDs. Given that our sample included staff from only two large EDs in Toronto, the results may not be generalizable to other hospital contexts, units, or roles. Future studies should expand the sample to diverse settings and incorporate triangulation methods such as integrating data from audits, drug monitoring logs, or direct observation, to validate perceptions and reduce reliance on self-reports alone. Finally, this study used two-mode network data and did not capture person-person relations but captured affiliations based on participation in a shared event (HCW-Task). As two-mode data was collected, traditional network analysis interpretations could not be used. Further research is needed to fully understand the social mechanisms (person-person relations) of diversion. We recommend that future studies should look to analyze suspected or confirmed cases of diversion within a patient-sharing network. This will provide a deeper understanding of the interprofessional connections involved in the ordering, administering, and reviewing of CS within the MUP and could potentially lead to more effective strategies for preventing diversion. Additionally, future research should aim to gather the perceptions of HCWs from a variety of hospitals, units, and roles. This approach would enhance the generalizability of participant responses, allowing for more comprehensive insights into understanding perceptions on in-hospital diversion.

## Conclusion

Using SNA, we identified that nurses in the ED play a central and influential role in tasks vulnerable to diversion, reflecting their extensive connectivity and involvement across the system. However, this centrality does not imply a greater likelihood of diversion by nurses but rather highlights their crucial position in processes that could be leveraged for diversion by others. The role of dedicated ED Pharmacists was highlighted as crucial in identifying discrepancies and safeguarding against diversion. We identified creating, following, or reviewing orders for patients not admitted (T1.1) as susceptible to diversion due to its frequent interprofessional connections and high vulnerability rating. While redundancy may help protect the network (e.g., enhance oversight), tasks like T1.1 remain vulnerable to diversion when HCWs act on decisions made by others. This emphasizes the importance of the initial ordering process and the need for technological and social mitigation efforts within the system. Mapping the network structure of each hospital unit can guide future interventions, such as altering the network shape (e.g., reshaping workflows), to strengthen ongoing efforts to prevent diversion.

## Supporting information

S1 Table Site 1 HCW centrality scores. (DOCX)

S2 Table Site 2 HCW centrality scores. (DOCX)

S3 Table Site 1 HCW redundancy scores. (DOCX)

S4 Table Site 2 HCW redundancy scores. (DOCX)
