# Supplementary material for: Unveiling hidden connections: How social networks impact diversion in hospital emergency departments: An exploratory social network analysis
Source: PLoS One. 2025 Sep 2;20(9):e0329176. doi: 10.1371/journal.pone.0329176 (PMC12404548; doi:10.1371/journal.pone.0329176)
Supplement: S2 Table — (DOCX) [file pone.0329176.s002.docx]

| **Node** | **Degree**  **(Normalized)** | **Eigenvector**  **(Normalized)** |
| --- | --- | --- |
| P1 | 0.13 | 0.04 |
| PA1 | 0.07 | 0.02 |
| PH1 | 0.13 | 0.03 |
| RN1 | 1.00 | 0.24 |
| RN2 | 1.00 | 0.24 |
| RN3 | 1.00 | 0.24 |
| RPN1 | 1.00 | 0.24 |
| RN4 | 1.00 | 0.24 |
| RN5 | 0.87 | 0.21 |
| RN6 | 0.87 | 0.22 |
| RN7 | 0.53 | 0.13 |
| RN8 | 0.80 | 0.20 |
| RN9 | 1.00 | 0.24 |
| RN10 | 1.00 | 0.24 |
| RN11 | 1.00 | 0.24 |
| RN12 | 1.00 | 0.24 |
| RN13 | 1.00 | 0.24 |
| RN14 | 1.00 | 0.24 |
| RN15 | 0.93 | 0.23 |
| RPN2 | 1.00 | 0.24 |
| RN16 | 1.00 | 0.24 |
| RN17 | 0.80 | 0.20 |
| NP1 | 0.07 | 0.02 |
| RN18 | 0.80 | 0.20 |
| RPN3 | 0.93 | 0.23 |
| RPN4 | 0.73 | 0.18 |
| RN19 | 1.00 | 0.24 |
| RN20 | 1.00 | 0.24 |
| P2 | 0.07 | 0.02 |
| P3 | 0.13 | 0.03 |
| P4 | 0.07 | 0.02 |
| P5 | 0.20 | 0.05 |
| P6 | 0.07 | 0.02 |
| P7 | 0.07 | 0.02 |
| P8 | 0.07 | 0.02 |
| P10 | 0.07 | 0.02 |
| P11 | 0.27 | 0.07 |
| P12 | 0.07 | 0.02 |
| P13 | 0.13 | 0.04 |
| P14 | 0.07 | 0.02 |
| P15 | 0.07 | 0.02 |
| P16 | 0.07 | 0.02 |
| P17 | 0.07 | 0.02 |
| RN22 | 1.00 | 0.24 |
| RN23 | 0.93 | 0.23 |
| RN24 | 1.00 | 0.24 |
| RN26 | 0.93 | 0.23 |
| RN27 | 0.87 | 0.21 |
| RN29 | 1.00 | 0.24 |
| RN30 | 1.00 | 0.24 |
| RN31 | 0.87 | 0.22 |
| RN33 | 1.00 | 0.24 |
| RN34 | 1.00 | 0.24 |
| RN36 | 1.00 | 0.24 |
| RN37 | 1.00 | 0.24 |
| RN38 | 1.00 | 0.24 |
| P18 | 0.07 | 0.02 |
