# Supplementary material for: Unveiling hidden connections: How social networks impact diversion in hospital emergency departments: An exploratory social network analysis
Source: PLoS One. 2025 Sep 2;20(9):e0329176. doi: 10.1371/journal.pone.0329176 (PMC12404548; doi:10.1371/journal.pone.0329176)
Supplement: S3 Table — (DOCX) [file pone.0329176.s003.docx]

| **Node** | **Redundancy** |
| --- | --- |
| RN1 | 105 |
| CNE1 | 106 |
| RN2 | 105 |
| RN3 | 105 |
| RN4 | 105 |
| RN5 | 105 |
| RN6 | 105 |
| RN7 | 105 |
| CC1 | 105 |
| RN8 | 105 |
| RN9 | 105 |
| RN10 | 105 |
| RN11 | 105 |
| RN12 | 105 |
| RN13 | 105 |
| RN14 | 105 |
| RN15 | 106 |
| RN16 | 105 |
| RPN1 | 107 |
| RN17 | 107 |
| RN18 | 105 |
| RN19 | 105 |
| RN20 | 105 |
| RN21 | 105 |
| RN24 | 105 |
| RN25 | 105 |
| RN26 | 106 |
| RN27 | 105 |
| RN28 | 105 |
| RN29 | 106 |
| RN30 | 105 |
| RN31 | 106 |
| RN32 | 105 |
| RN33 | 106 |
| RN34 | 105 |
| RN35 | 105 |
| RN36 | 105 |
| RN37 | 105 |
| RN38 | 105 |
| RN39 | 105 |
| RN40 | 105 |
| CN1 | 105 |
| RN41 | 105 |
| RN42 | 105 |
| RN43 | 105 |
| RN44 | 105 |
| RN45 | 106 |
| RN46 | 105 |
| CN2 | 105 |
| RN47 | 106 |
| RN48 | 105 |
| RN49 | 105 |
| RN50 | 105 |
| RN51 | 105 |
| RN52 | 105 |
| P1 | 112 |
| P2 | 112 |
| P3 | 112 |
| PM1 | 102 |
