# Supplementary material for: Unveiling hidden connections: How social networks impact diversion in hospital emergency departments: An exploratory social network analysis
Source: PLoS One. 2025 Sep 2;20(9):e0329176. doi: 10.1371/journal.pone.0329176 (PMC12404548; doi:10.1371/journal.pone.0329176)
Supplement: S4 Table — (DOCX) [file pone.0329176.s004.docx]

| **Node** | **Redundancy** |
| --- | --- |
| PA1 | 68 |
| PH1 | 87 |
| RN1 | 71.6 |
| RN2 | 71.6 |
| RN3 | 71.6 |
| RPN1 | 71.6 |
| RN4 | 71.6 |
| RN5 | 72.6 |
| RN6 | 73.1 |
| RN7 | 74.2 |
| RN8 | 73.8 |
| RN9 | 71.6 |
| RN10 | 71.6 |
| RN11 | 71.6 |
| RN12 | 71.6 |
| RN13 | 71.6 |
| RN14 | 71.6 |
| RN15 | 71.9 |
| RPN2 | 71.6 |
| RN16 | 71.6 |
| RN17 | 73.5 |
| NP1 | 66 |
| RN18 | 73.5 |
| RPN3 | 72.6 |
| RPN4 | 74.4 |
| RN19 | 71.6 |
| RN20 | 71.6 |
| RN22 | 71.6 |
| RN23 | 72.3 |
| RN24 | 71.6 |
| RN26 | 72.6 |
| RN27 | 69.2 |
| RN29 | 71.6 |
| RN30 | 71.6 |
| RN31 | 73.4 |
| RN33 | 71.6 |
| RN34 | 71.6 |
| RN36 | 71.6 |
| RN37 | 71.6 |
| RN38 | 71.6 |
| P1 | 91 |
| P2 | 106 |
| P3 | 87 |
| P4 | 106 |
| P5 | 80 |
| P6 | 106 |
| P7 | 106 |
| P8 | 106 |
| P10 | 106 |
| P11 | 77.5 |
| P12 | 106 |
| P13 | 91 |
| P14 | 106 |
| P15 | 106 |
| P16 | 106 |
| P17 | 106 |
| P18 | 106 |
